# Supplementary material for: Differentiating Mpox Infection and Vaccination Using a Validated Multiplex Orthopoxvirus IgG Serology Assay
Source: medRxiv. 2025 Oct 27:2025.10.24.25338410. Preprint. [Version 1] doi: 10.1101/2025.10.24.25338410 (PMC12636651; doi:10.1101/2025.10.24.25338410)
Supplement: 1 [file NIHPP2025.10.24.25338410V1-supplement-1.pdf]

medRxiv preprint doi: https://doi.org/10.1101/2025.10.24.25338410; this version posted October 27, 2025. The copyright holder for this preprint (which was not certified by peer review) is the author/funder, who has granted medRxiv a license to display the preprint in perpetuity.
It is made available under a CC-BY 4.0 International license .

Supplementary Material

Validation

Analytical sensitivity

The manufacturer does not specify a fixed limit of detection (LoD) for the VPLEX Orthopoxvirus assay, and instead the LoD is calculated for each run of the assay. The default method for calculating the LoD in the MSD Discovery Workbench 4.0 software utilizes the CAL-08 Signal results as follows:

$$LoD = mean_{control} \times 2.5 \times SD_{control}$$; control is the specimen diluent alone (CAL-08)

Once the LoD is calculated, in terms of Signal, it is converted to AU/mL utilizing the calibrator curve. To establish a fixed LoD, calibrators were run in duplicate over ten days (Figure S1A for results in Signal and S1B for results in AU/mL). Comparison of Signal results for each of the calibrators over time were distinct across all calibrators for antigens MPXV A29L, VACV A27L, MPXV A35R, VACV A33R, and VACV D8L. In contrast, antigens MPXV B6R, VACV B5R, MPXV E8L, MPXV M1R, and VACV L1R there was overlap in Signal between calibrators over time, especially the lower concentration calibrators and the blank (CAL-05 thru CAL-07 and CAL-08). Standardization to AU/mL largely resolves this overlap since it normalizes Signal differences between runs except for CAL-07 and CAL-08 for some antigens (MPXV M1R and VACV L1R). The LoD was determined for each antigen using the MSD Discovery Workbench software over the ten runs and the geometric mean of the LoD for each antigen ranged from 0.0031 – 0.0038 AU/mL. Thus, we set the LoD for each antigen at 0.004 AU/mL (Table S1)

For quality control an upper limit was set for CAL-08 of a Signal of 300 based on the range of values observed during our validation runs (Figure S2 – validation runs annotated with light blue bar). Notably, during validation, four out of ten runs exhibited higher CAL-08 Signal relative to the other runs for the MPXV M1R and VACV L1R antigens. For these antigens, the percent relative error (%RE) of the control materials provided by the manufacture were within +/- 30% for control 1 and 2 and < 0.2 AU/mL for control 3 and the geometric standard deviation of the control materials were all within 1.42. Thus, despite the slightly elevated CAL-08 Signal the assays were considered valid. During subsequent testing, several assays were flagged for having CAL-08 Signal exceeding a Signal of 300, especially for the MPXV M1R and VACV L1R antigens (Figure S2 between Oct 2024 and Jan 2025). Again, the manufactures controls performed as expected based on criteria described above. Plate images and data were provided to the manufacturer, and the manufacturer could not identify any artifacts (e.g. well drying) or manufacturing correlates that could explain the elevated CAL-08 Signal. All assays were performed by two operators using the same lot number of materials. To ensure sufficient dynamic range in Signal, the criteria was changed to ensure at least a 200-fold difference between CAL-01 and CAL-08 signals, rather than utilizing an absolute value for CAL-08 Signal. Based on this criterion,

medRxiv preprint doi: https://doi.org/10.1101/2025.10.24.25338410; this version posted October 27, 2025. The copyright holder for this preprint (which was not certified by peer review) is the author/funder, who has granted medRxiv a license to display the preprint in perpetuity.
It is made available under a CC-BY 4.0 International license .

out of 75 assays, four assays failed for the M1R antigen and one assay failed for both M1R and L1R antigen (Figure S2, red-filled points).

Verification of Calibration model

The calibration model was verified by testing of the calibrators over ten days. The day-to-day percent relative error was within +/- 30% for all calibrators, except CAL-07 which was inconsistent from day-to-day for some antigens (data not shown). The average percent relative error observed over ten days of testing was within +/- 15% (Figure S3A). Finally, within-lab imprecision was within 15% geometric coefficient of variation (GCV) for all calibrators, except CAL-07 which was greater than GCV of 15% for a subset of the antigens (Figure S3B). Lastly, the four-parameter logistic regression (4PL) fits for each antigens calibration curve had R^2 values > 0.9 (Table S2). Taken together, these data verify the validity of the calibration model and that CAL-07.

Based on the imprecision of the calibrators shown here the lower limit of quantification (LLoQ), was less than the LLoQ reported by the manufacturer (Table S1). The more conservative manufacturers estimates were selected as the preliminary LLoQ (see Table S1, LLoQ final column). The ULoQ was set to the mean back-calculated AU/mL value of the highest calibrator (CAL-01) (see Table S1, ULoQ final avg. calculated AU/mL of CAL-01).

Linearity

To assess linearity the results within the limits of quantification (LoQ) from testing of the assay calibrators over ten days were subjected to linear regression analysis (Figure S4A). For all antigens, the calibrators demonstrated strong linearity with R^2 values exceeding 0.9. Furthermore, for all antigens, the slopes of the linear regression were approximately one, and the y-intercepts were below 0.2 AU/mL, indicating the absence of systemic or proportional bias.

We also assessed linearity with strongly positive serum specimen (M32) that was previously tested in the V-PLEX Orthopoxvirus assay. The specimen was initially diluted 50-fold followed by seven 4-fold dilutions. Each dilution was measured in the assay and the back-calculated AU/mL values, not corrected for dilution, were subjected to linear regression (Figure S4B). Like the calibrators, the M32 dilution series was strongly linear with R^2 values exceeding 0.9. Furthermore, for all antigens, the slopes of the linear regression were approximately one, and the y-intercepts were below 0.2 AU/mL, indicating the absence of systemic or proportional bias. Lastly, the M32 dilution series results were highly parallel to the calibrator linear regression (Figure S4B, dotted line corresponds to the calibrator linear regression best fit), supporting the use of the calibrators and selected diluent in estimating AU/mL levels from serum specimens. Similar results were obtained with Reference Vaccinia Immune Globulin (VIGIG) pooled human serum from CBER/FDA lot 1 (data not shown).

medRxiv preprint doi: https://doi.org/10.1101/2025.10.24.25338410; this version posted October 27, 2025. The copyright holder for this preprint (which was not certified by peer review) is the author/funder, who has granted medRxiv a license to display the preprint in perpetuity.
It is made available under a CC-BY 4.0 International license .

Precision

Assay imprecision was estimated from two data sets. First, two human serum specimens from MPXV-infected subjects and three human serum specimens from Jynneos-vaccinated subjects were selected. Each specimen was tested in duplicate, with each replicate tested utilizing two wells, across six separate days (Figure S5A). These specimens spanned a broad range of antibody concentrations within the assay's quantifiable limits, as determined by calibrator performance. Second, results from testing the manufacturer supplied serology controls (1,2, and 3), with two technical replicates (two wells), over ten days were also analyzed (Figure S5B). Collectively, the three controls span the assay's quantitative range. Control 3 fell just below the lower limit of quantification (LLoQ) for MPXV A29L, VACV A27L, and MPXV B6R antigens, which is expected since control 3 is presumed negative. Variance decomposition was performed using ANOVA to estimate intra-assay, inter-assay, and within-laboratory components. Results with an inter-well geometric coefficient of variation (GCV) > 37% were excluded, as this level of variability between technical replicates may indicate an unreliable measurement. Only human serum specimen V4 was affected, with one VACV L1R result (day 1) and one MPXV M1R result (day 10) removed. All human serum specimens demonstrated within-laboratory imprecision with GCV <37% (Figure S6A). For the serology controls, despite some control 3 results falling below the LLoQ, all three controls exhibited within-laboratory imprecision with GCV < 37% (Figure S6B). Notably, the serology controls showed lower and more consistent imprecision compared to the selected specimens described above, so they don't appear to entirely reflect real-world variability.

Accuracy

Accuracy was evaluated by comparing measured results from the manufacturer-supplied serology controls (Control 1, Control 2, and Control 3) to their manufacturer assigned AU/mL values. Control 1 and Control 2 are expected to fall within the high and mid quantitative ranges of each assay (Figure S5B). Each control was tested in duplicate wells across ten independent runs. For both Control 1 and Control 2, the relative error compared to the manufacturer-assigned AU/mL values was within ±30% for all assays (Figure S7A). Control 3 serves as a presumptive negative control, with assigned AU/mL values of < 0.1 AU/mL for VACV A27L, MPXV A29L, VACV A33R, MPXV A35R, VACV B5R, MPXV B6R, VACV D8L, and MPXV E8L, and < 0.2 AU/mL for VACV L1R and MPXV M1R. Control 3 was tested alongside the other controls in duplicate wells across ten runs (Figure S5B). The geometric mean response and geometric standard deviation (GSD) were calculated, and an empirical limit for Control 3 was determined by adding three times the GSD to the geometric mean (limits shown in red in Figure S7B). All empirically determined limits for Control 3 were ≤ 0.1 AU/mL. Together, these results demonstrate the accuracy of the assay relative to the manufacturer-supplied serology controls (Controls 1–3) since the percent relative error of control 1 and 2 are < ± 30% and Control 3 AU/mL values were ≤ 0.1 AU/mL.

medRxiv preprint doi: https://doi.org/10.1101/2025.10.24.25338410; this version posted October 27, 2025. The copyright holder for this preprint (which was not certified by peer review) is the author/funder, who has granted medRxiv a license to display the preprint in perpetuity.
It is made available under a CC-BY 4.0 International license .

Analytical Measurement Range

The lower limit of quantification (LLoQ) was defined as the minimum concentration above the limit of detection (LoD) that meets precision criteria and falls within the assay's linear range. The upper limit of quantification (ULoQ) was defined as the maximum concentration that meets precision criteria and remains within the linear range. Together, the LLoQ and ULoQ establish the analytical measurement range (AMR) of the assay. All calibrator-derived results within the LLoQ and ULoQ met acceptance criteria for both linearity (Figure S4) and imprecision (Figures S5 and S6). Therefore, the LLoQ and ULoQ values presented in Table S1 define the assay's validated AMR.

Robustness

Since clinical specimens are likely to undergo several freeze thaws (FT) between collection and testing, the effect of FT on the assay results was evaluated. Four specimens were selected, two MPXV-infected (M42, M48) and two smallpox vaccinated (V14, V57). Each specimen was thawed and four aliquots prepared and frozen back down. One tube was designated as freeze-thaw (FT) 0 and not thawed again till testing, one tube was designated as FT 1 and was thawed once and frozen down again prior to testing, one tube was designated as FT 2 and was thawed and frozen twice prior to testing, one tube was designated as FT 3 and was thawed and frozen three times prior to testing. The ratio of antibody level results between FT 0 and each of the different freeze-thaw specimens (1-3), was calculated and all ratios were within 0.5 -1.5-fold, so no significant freeze-thaw effects were detected (Figure S8).

medRxiv preprint doi: https://doi.org/10.1101/2025.10.24.25338410; this version posted October 27, 2025. The copyright holder for this preprint (which was not certified by peer review) is the author/funder, who has granted medRxiv a license to display the preprint in perpetuity.
It is made available under a CC-BY 4.0 International license .

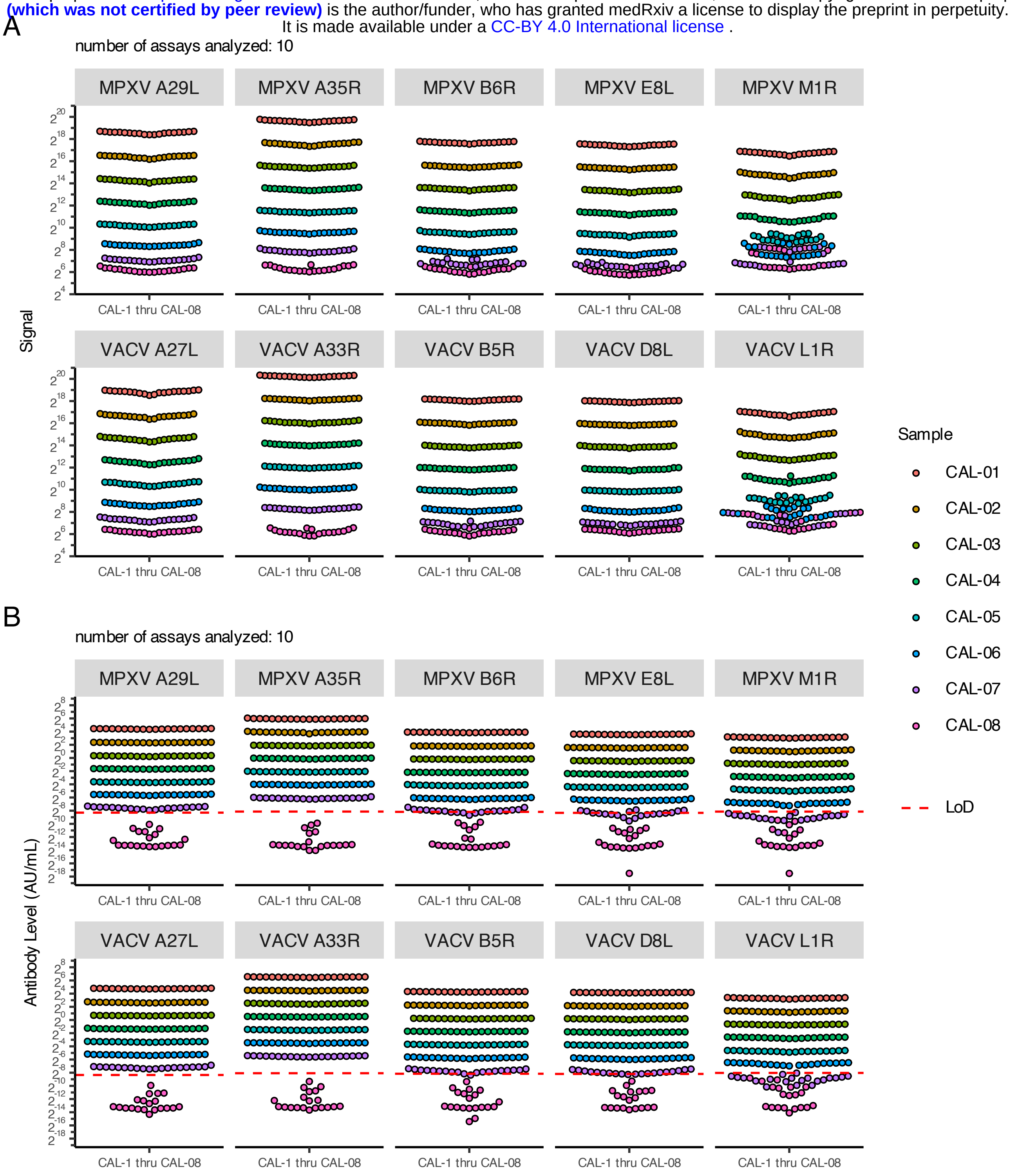

Figure S1: Signal results from different days overlap among the lowest calibrators, but conversion to AU/mL normalizes for the day-to-day differences in Signal. Shown are calibrator results from ten independent runs of the MSD Orthopoxvirus assay in units of Signal (A) and antibody level in AU/mL (B). For MPXV A29L, VACV A27L, MPXV A35R, VACV A33R antigens CAL-01 thru CAL-08 are entirely distinguishable by Signal even between runs on different days. However, the remaining antigens showed some degree of Signal overlap between CAL-05 thru CAL-08. Examination of Signal levels within any given run showed that the Signal levels are distinguishable for CAL-01 thru CAL-07 for each of the antigens. Conversion of Signal to AU/mL normalizes results such that overlap is limited to CAL-07 and CAL-08. The limit of detection (LoD) for each antigen for each run was calculated following the described manufactures recommendation and the average of these results is indicated by the red dashed line. When the CAL-08 signal fell below the lower asymptote of the 4PL model used to convert Signal to AU/mL, an AU/mL value could not be calculated and was arbitrarily set to the lowest reportable value based on the 4PL model fit.

medRxiv preprint doi: https://doi.org/10.1101/2025.10.24.25338410; this version posted October 27, 2025. The copyright holder for this preprint (which was not certified by peer review) is the author/funder, who has granted medRxiv a license to display the preprint in perpetuity.
It is made available under a CC-BY 4.0 International license .

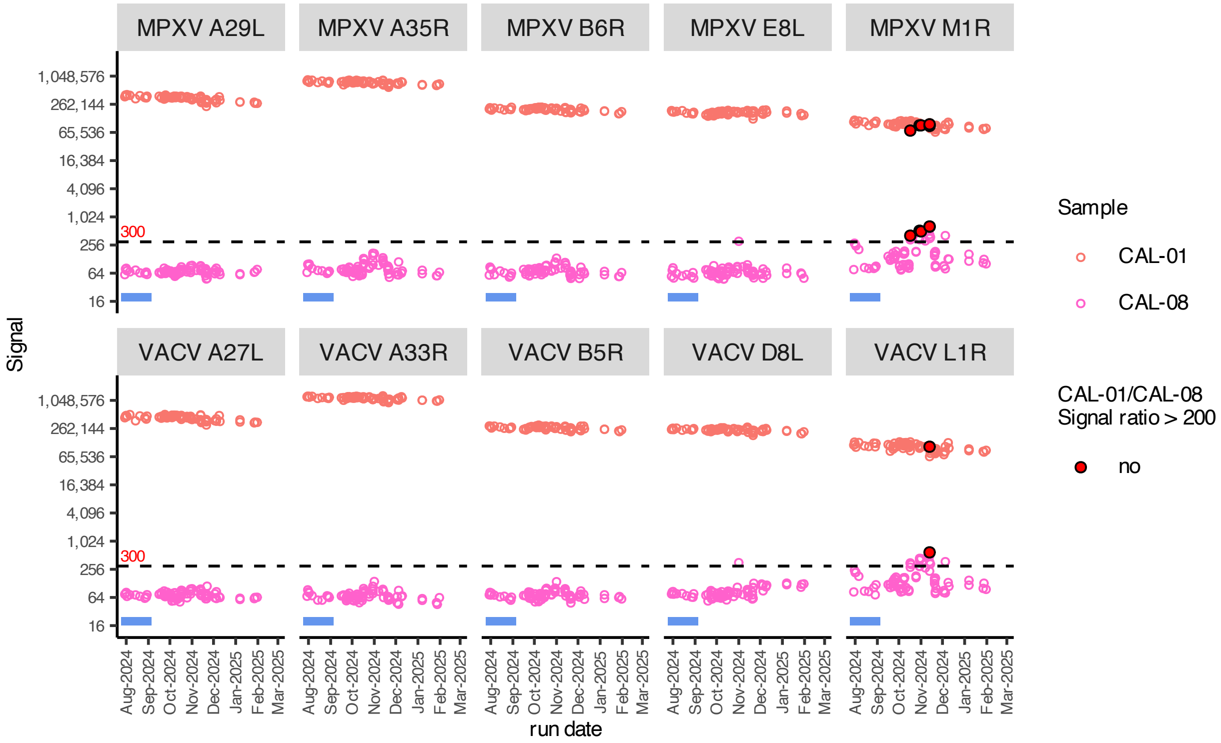

Figure S2: Greater variation in CAL-08 Signal over time is evident for antigens MPXV M1R and VACV L1R. Shown are CAL-01(red) and CAL-08 (purple) Signal values both during validation of the assay (see light-blue shaded bars) and subsequent testing over the following 5-6 months. The dashed blank line indicates the initial quality control threshold that CAL-08 was expected to not exceed. Red filled points are assays that did not meet CAL-01/CAL-08 Signal ratio of at least 200, which included five M1R assays and one L1R assay.

medRxiv preprint doi: https://doi.org/10.1101/2025.10.24.25338410; this version posted October 27, 2025. The copyright holder for this preprint (which was not certified by peer review) is the author/funder, who has granted medRxiv a license to display the preprint in perpetuity.
It is made available under a CC-BY 4.0 International license .

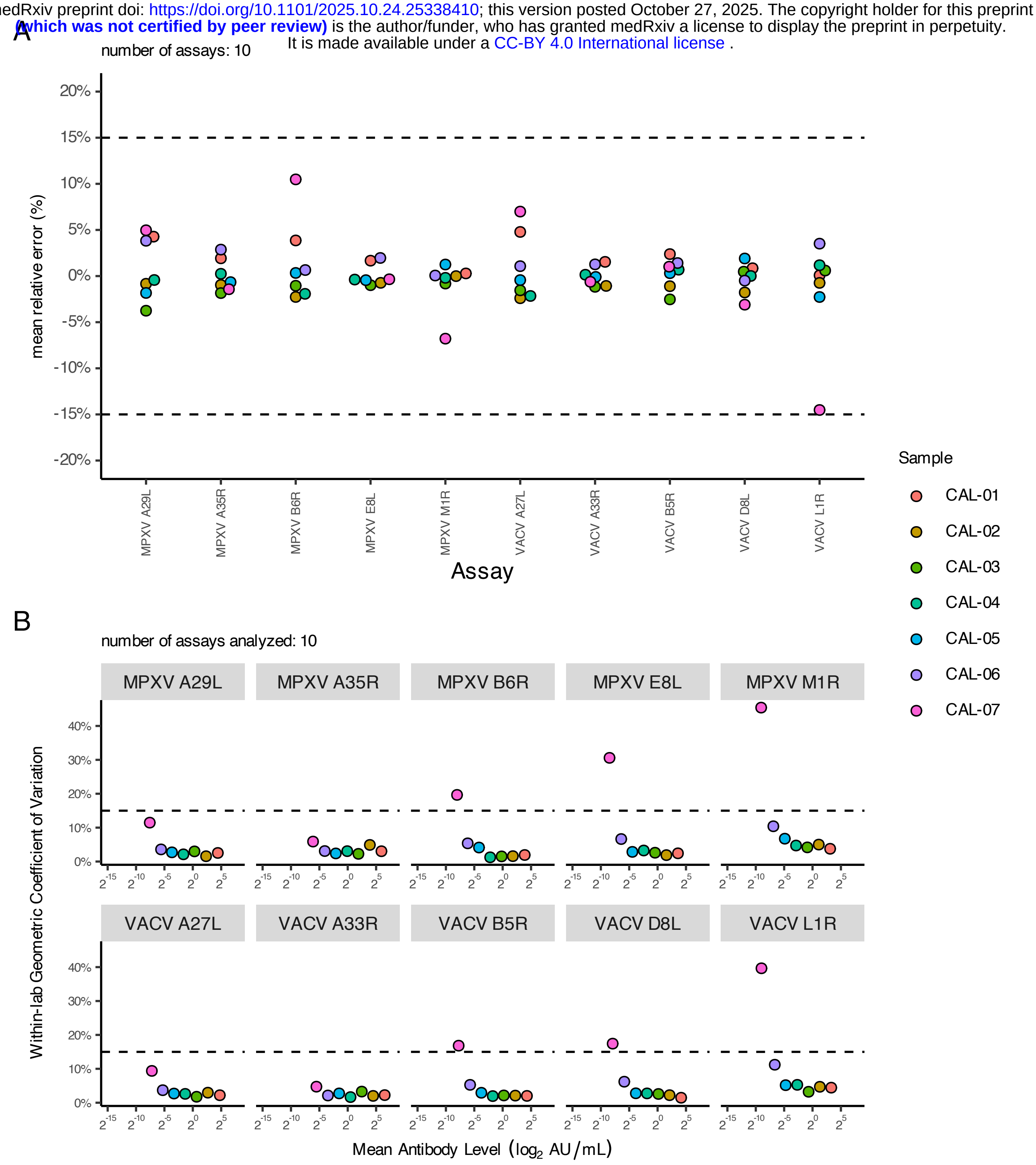

Figure S3: Average relative error of calibrators is within +/- 15 and within-lab geometric coefficient of variation of each calibrator is within 15% for CAL-01 thru CAL-06. Assay calibrators (CAL-01 thru CAL-07) were tested in duplicate over ten runs (A) Shown is the mean percent relative error for each calibrator (CAL-01 thru CAL-07) with the acceptability criteria of mean relative error of +/- 15% indicated by black dashed line. The mean relative error estimates are based on AU/mL values. (B) From the same data the within-lab geometric coefficient of variation (GCV) was calculated and plotted for each calibrator. Dashed black lines mark the acceptability criteria of a within-lab GCV of 15% or lower. Both the mean relative error estimates and the %GCV is calculated from AU/mL values.

medRxiv preprint doi: https://doi.org/10.1101/2025.10.24.25338410; this version posted October 27, 2025. The copyright holder for this preprint (which was not certified by peer review) is the author/funder, who has granted medRxiv a license to display the preprint in perpetuity.
It is made available under a CC-BY 4.0 International license .

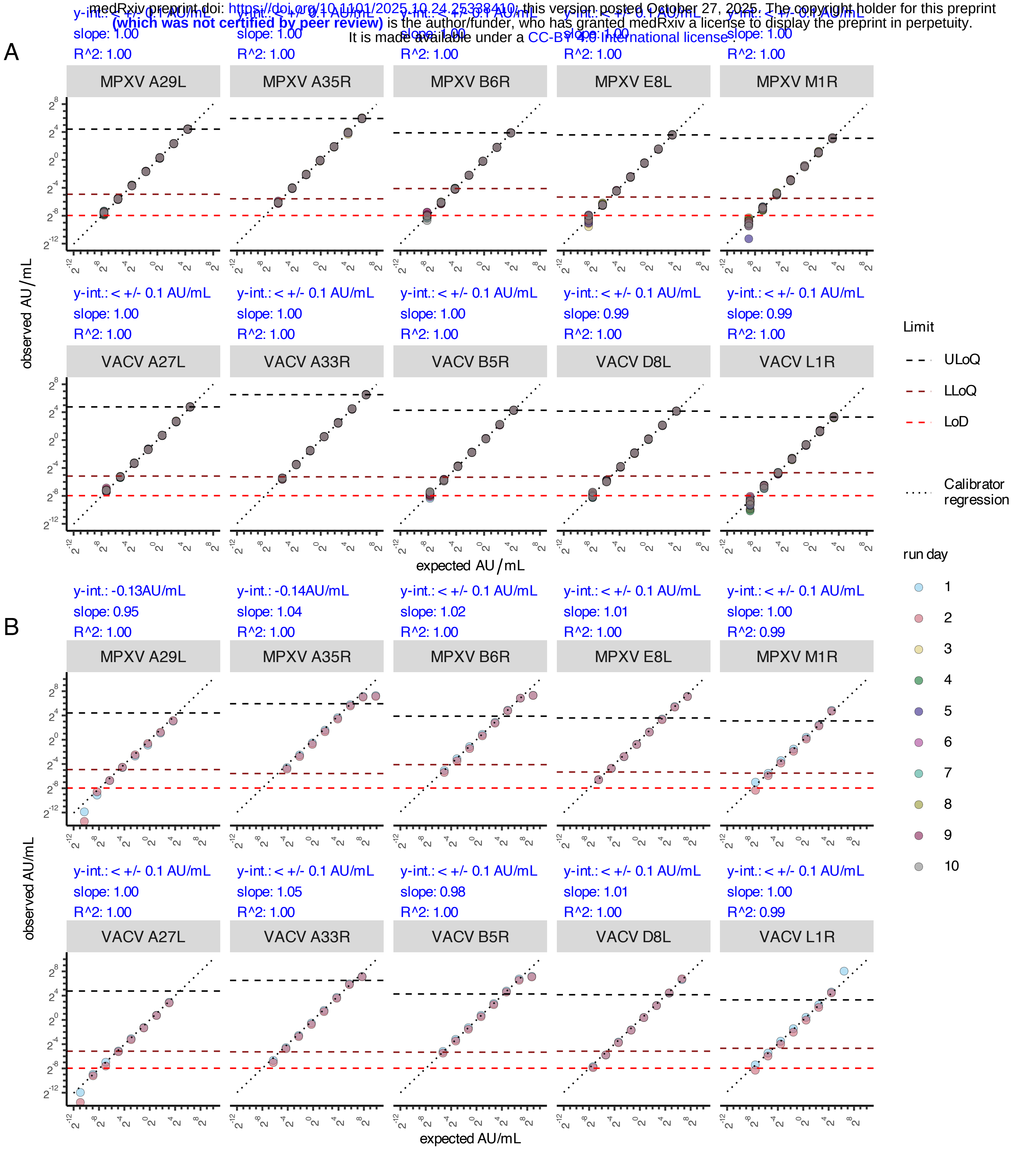

Figure S4: Antibody levels in the quantitative range of the MSD Orthopoxvirus assay are strongly linear. Linear regression was performed against observed (y-axis) and expected (x-axis) antibody levels (AU/mL) from testing (A) assay calibrators over ten days or (B) serially diluted serum specimen from MPXV-infected individual over two days (M32). Only results within the lower and upper limits of quantification were considered for linear regression (see Table S1). The expected AU/mL values for the calibrator are based on values provided by the manufacturer and the expected AU/mL values for the M32 specimen were based on testing of the M32 specimen in the MSD Orthopoxvirus assay at a dilution of 1/5000. The y-intercept, slope and R2 value determined from each linear regression is summarized in blue text above each plot. The dotted line represents the best fit line based on the linear regression of the calibrators only. The LoD, LLoQ, and ULoQ for each antigen are indicated in red, dark red, and black dashed lines, respectively.

medRxiv preprint doi: https://doi.org/10.1101/2025.10.24.25338410; this version posted October 27, 2025. The copyright holder for this preprint
(which was not certified by peer review) is the author/funder, who has granted medRxiv a license to display the preprint in perpetuity.
It is made available under a CC-BY 4.0 International license .

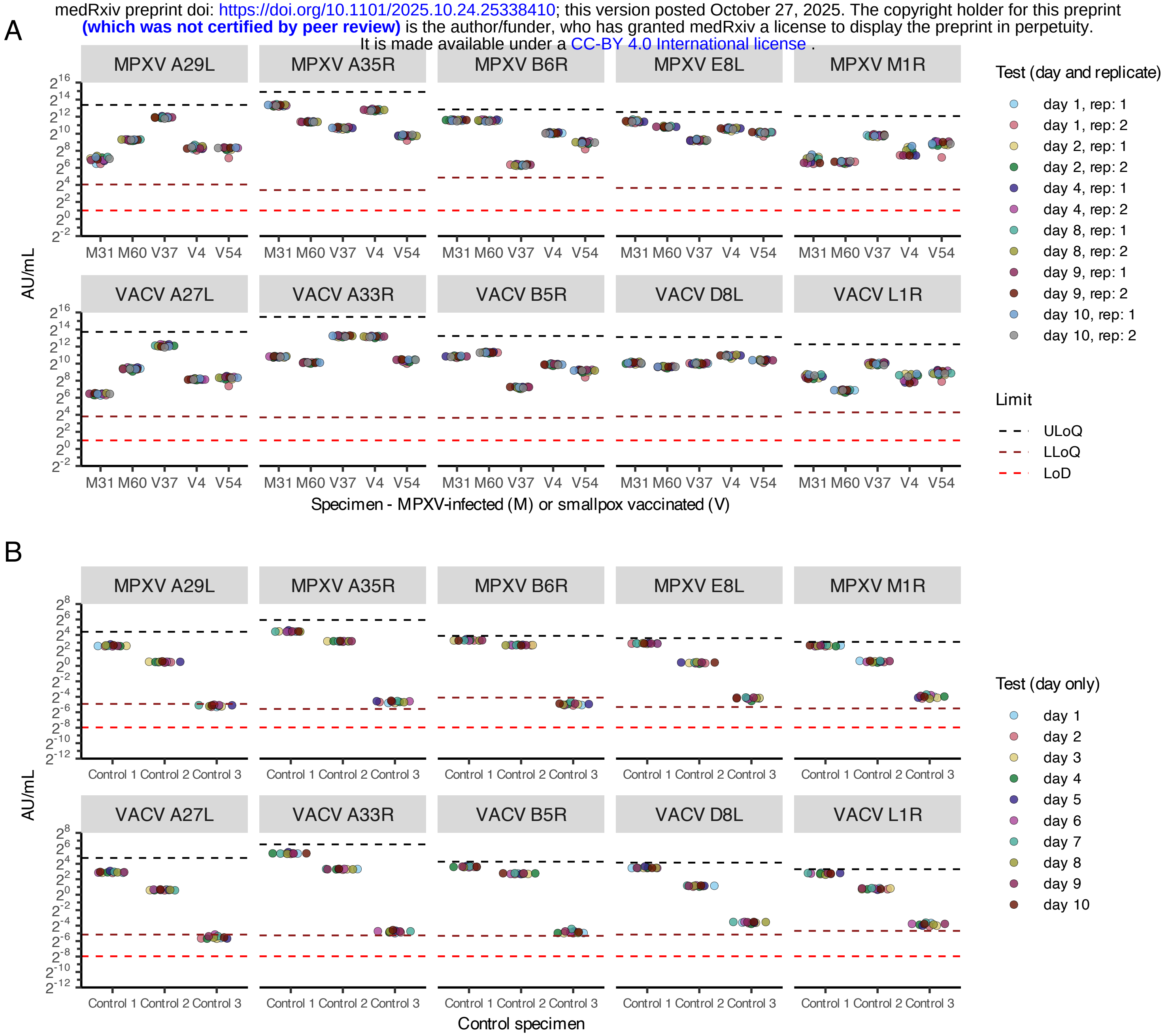

Figure S5: Antibody level (AU/mL) results for estimation of assay imprecision from either human serum specimens or serology controls provided by the manufacturer span the quantitative range of the assay. (A) Two serum specimens from MPXV-infected subjects (M) and three serum specimens from vaccinated subjects (V), which represent a range of antibody levels in the assay were selected for assessing assay imprecision. Specimens were tested over six days in duplicate, with reach replicate tested with two technical replicates (i.e. two wells per replicate) for a total of twelve average results per specimen. (B) Assay controls were tested once per run and the ten results shown are the average response from two wells. The ULoQ (black dashed line), LLoQ (brown dashed line), and LoD (red dashed line) established based on calibrator performance are shown. The LoD, LLoQ, and ULoQ are higher in (A) than (B) since human serum specimens were tested at a 500-fold dilution, whereas the serology controls were tested neat.

medRxiv preprint doi: https://doi.org/10.1101/2025.10.24.25338410; this version posted October 27, 2025. The copyright holder for this preprint (which was not certified by peer review) is the author/funder, who has granted medRxiv a license to display the preprint in perpetuity.
It is made available under a CC-BY 4.0 International license .

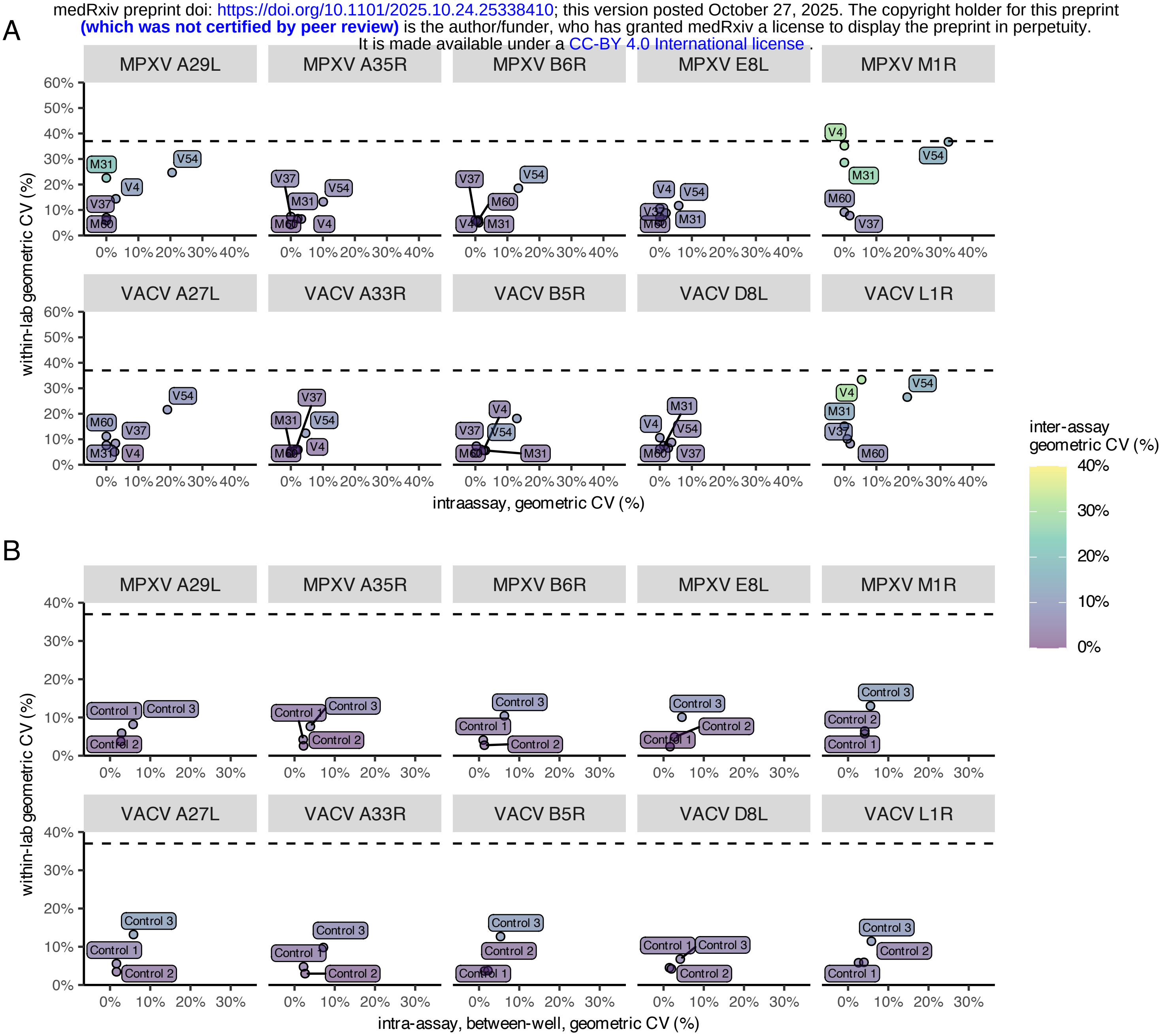

Figure S6: Imprecision of human serum specimens and serology controls meet acceptance criteria of within-lab geometric coefficient of variation (CV) of < 37%. Based on the testing shown Figure S5, variance decomposition using ANOVA was performed to determine within-lab (y-axis), inter-assay (coloring of labels and points), and intra-assay imprecision (x-axis). (A) Are the imprecision results from testing human serum specimens shown in Figure S5A. Specimens IDs include whether there are from individuals MPXV-infected (M) or smallpox vaccinated (V). For specimen V4, a day 1 VACV L1R result and a day 10 MPXV M1R result, both with between-well geometric CV of greater than 37%, were removed for this analysis. (B) Are the imprecision results from testing the serology controls. Since the serology controls were only tested once, with two technical replicates, the intraassay imprecision is between-well.

medRxiv preprint doi: https://doi.org/10.1101/2025.10.24.25338410; this version posted October 27, 2025. The copyright holder for this preprint (which was not certified by peer review) is the author/funder, who has granted medRxiv a license to display the preprint in perpetuity.
It is made available under a CC-BY 4.0 International license .

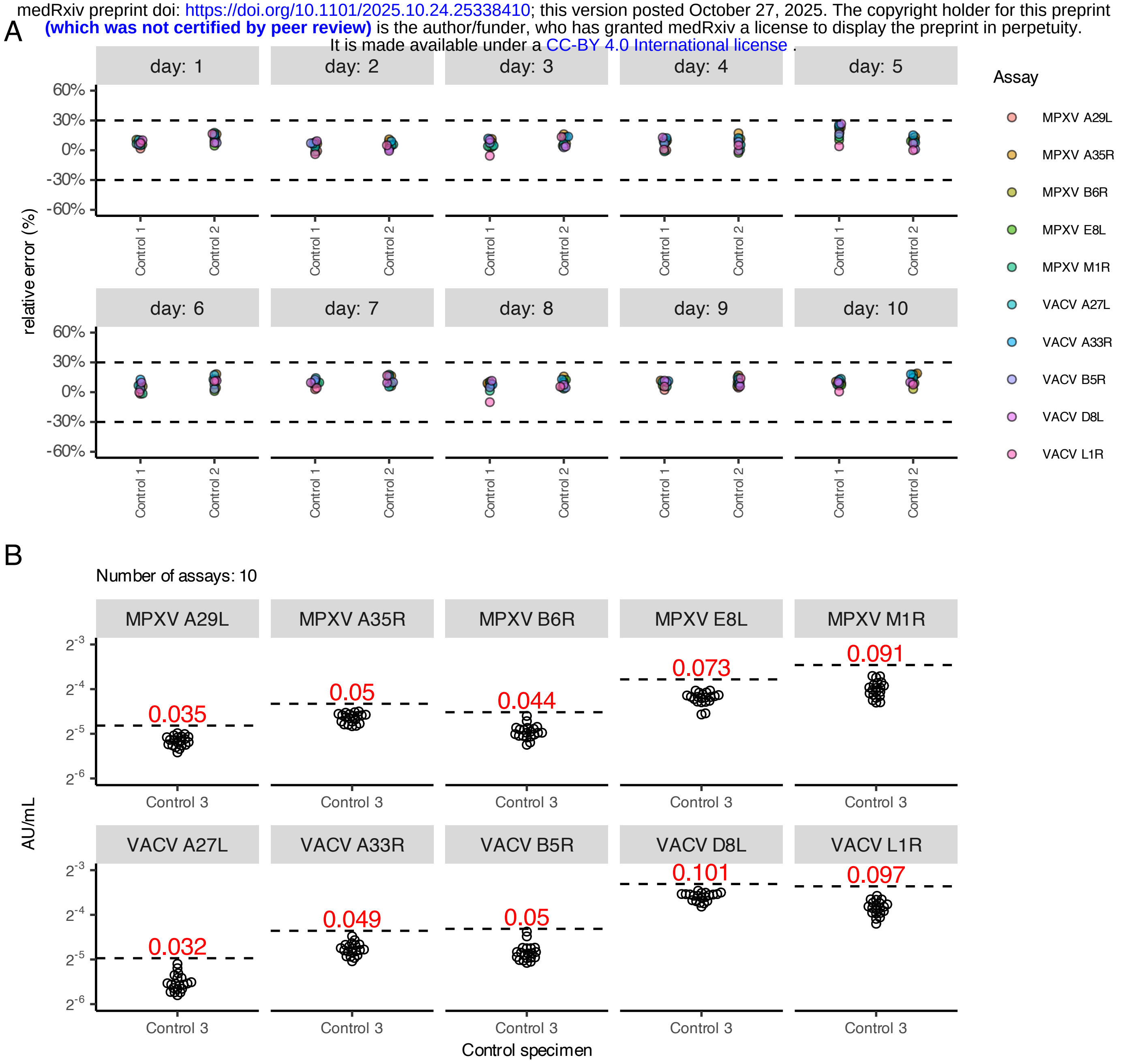

Figure S7: Based on manufacturer supplied serology controls the MSD Orthopoxvirus assay is accurate given that day-to-day relative error for Control 1 and 2 is less than +/- 30% and control 3 limit antibody levels are all less than 0.2 AU/mL. Serology controls (1,2 and 3) provided by the manufacturer were tested with two technical replicates over ten days. (A) Shown are the results for serology controls 1 and 2. The dashed black lines mark the acceptance criteria of a relative error of +/- 30%. (B) Shown are the results for serology control 3. The dashed black line is set to the control 3 geometric mean plus three times the control 3 geometric standard deviation. The acceptance criterion is meet since the Control 3 limit is less than 0.2 AU/mL for each antigen.

medRxiv preprint doi: https://doi.org/10.1101/2025.10.24.25338410; this version posted October 27, 2025. The copyright holder for this preprint
(which was not certified by peer review) is the author/funder, who has granted medRxiv a license to display the preprint in perpetuity.
It is made available under a CC-BY 4.0 International license .

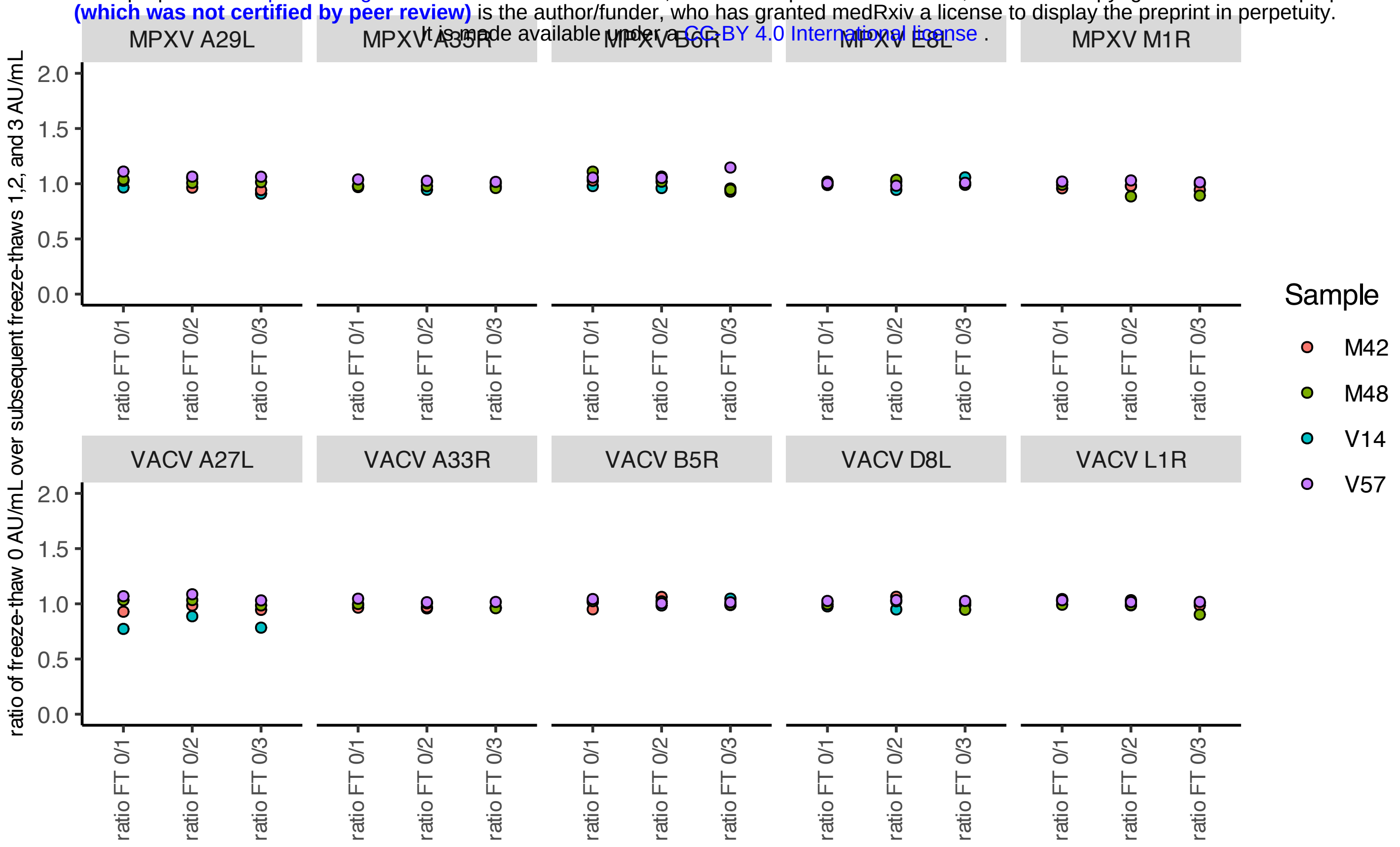

Figure S8: The assay is robust against freeze-thaw cycles of serum specimens. Plotted is the AU/mL ratio of the results before freeze-thaw (FT 0) over results from freeze-thaw 1 (ratio FT 0/1), from freeze-thaw 2 (ratio FT 0/2), or from freeze-thaw 3 (ratio FT 0/2). Shown are results from testing four specimens and specimens IDs include whether there are from individuals MPXV-infected (M) or smallpox vaccinated (V).

medRxiv preprint doi: https://doi.org/10.1101/2025.10.24.25338410; this version posted October 27, 2025. The copyright holder for this preprint (which was not certified by peer review) is the author/funder, who has granted medRxiv a license to display the preprint in perpetuity.
It is made available under a CC-BY 4.0 International license.

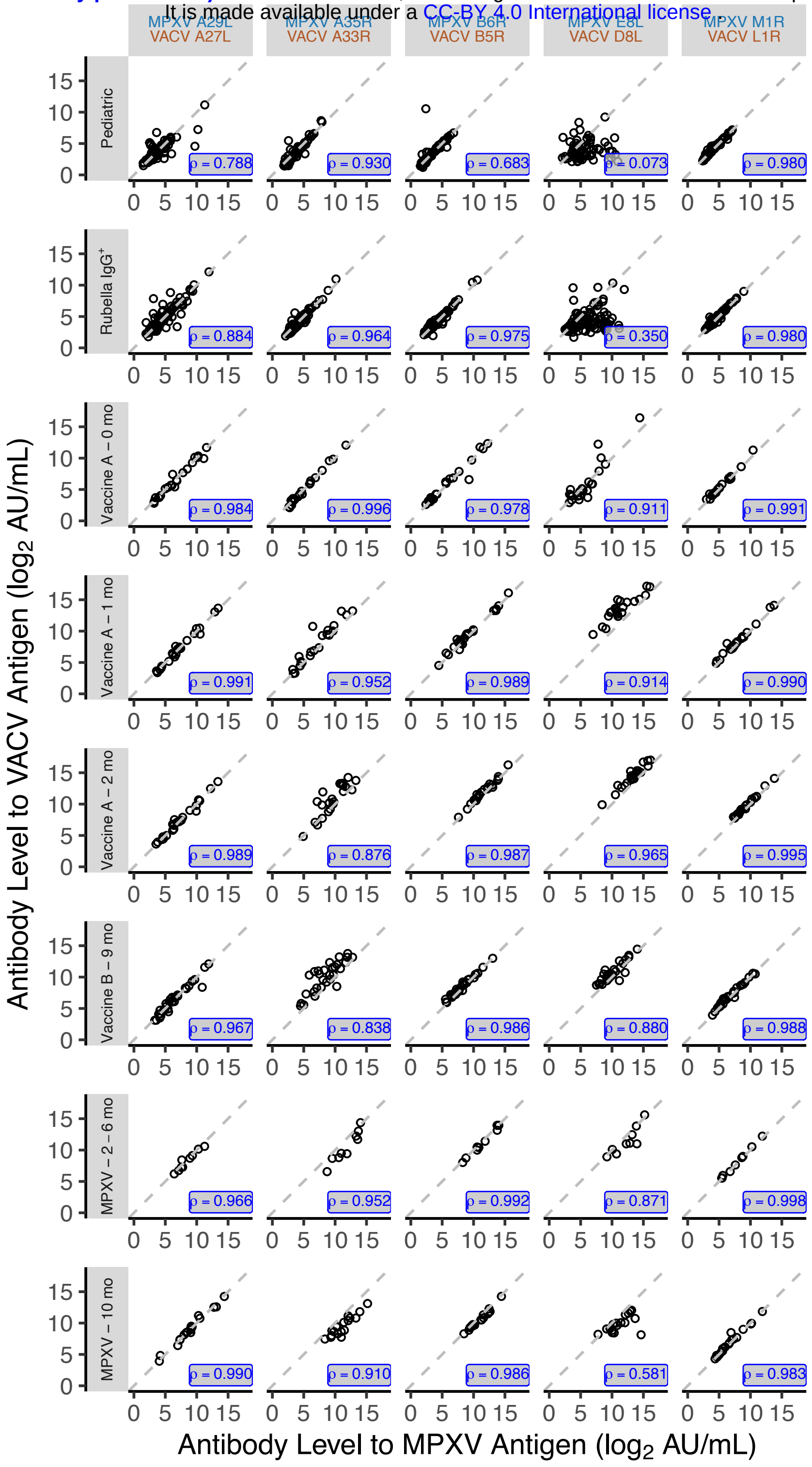

Figure S9: Antibody titer results against antigen orthologs are highly correlated. Shown are pairwise plots of the log2 transformed AU/mL results between antigen ortholog pairs within in each cohort. Inset value in blue is the Pearson's correlation coefficient, also shown in Figure 1A. The P61 MPXV B6R/VACV B5R results were omitted from the plot, since the VACV B5R antibody level was undetected.

medRxiv preprint doi: https://doi.org/10.1101/2025.10.24.25338410; this version posted October 27, 2025. The copyright holder for this preprint (which was not certified by peer review) is the author/funder, who has granted medRxiv a license to display the preprint in perpetuity.
It is made available under a CC-BY 4.0 International license .

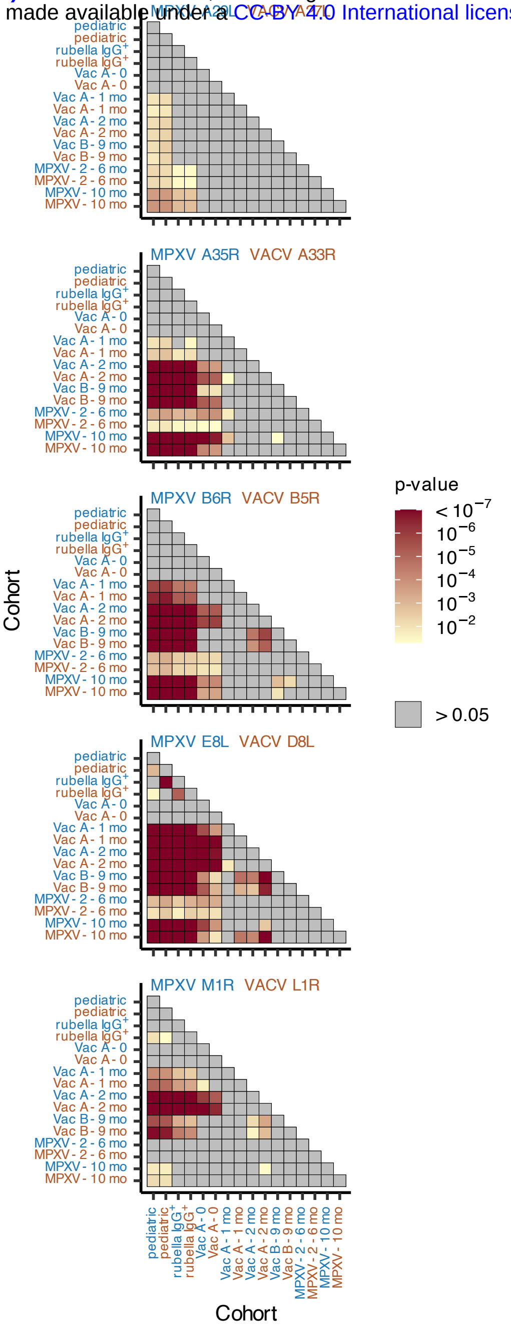

Figure S10. Significant differences in anti-orthopoxvirus antibody levels among MPXV-infected, vaccinated, and negative control cohorts. For each MPXV and VACV antigen pair (indicated along the top of each matrix), log-transformed antibody levels (AU/mL) were compared between cohorts using two-sided t-tests. Cohort names are color-coded: blue for MPXV antigen results and brown for VACV antigen results. The p-values are displayed in a lower-triangular matrix, where each tile represents a pairwise comparison as indicated by the intersecting column and row names. Tile shading intensity is inversely proportional to the p-value (i.e. darker red corresponds to a smaller p-value); gray tiles indicate p-value > 0.05. All p-values are Bonferroni-adjusted for multiple comparisons.

medRxiv preprint doi: https://doi.org/10.1101/2025.10.24.25338410; this version posted October 27, 2025. The copyright holder for this preprint
(which was not certified by peer review) is the author/funder, who has granted medRxiv a license to display the preprint in perpetuity.
It is made available under a CC-BY 4.0 International license .

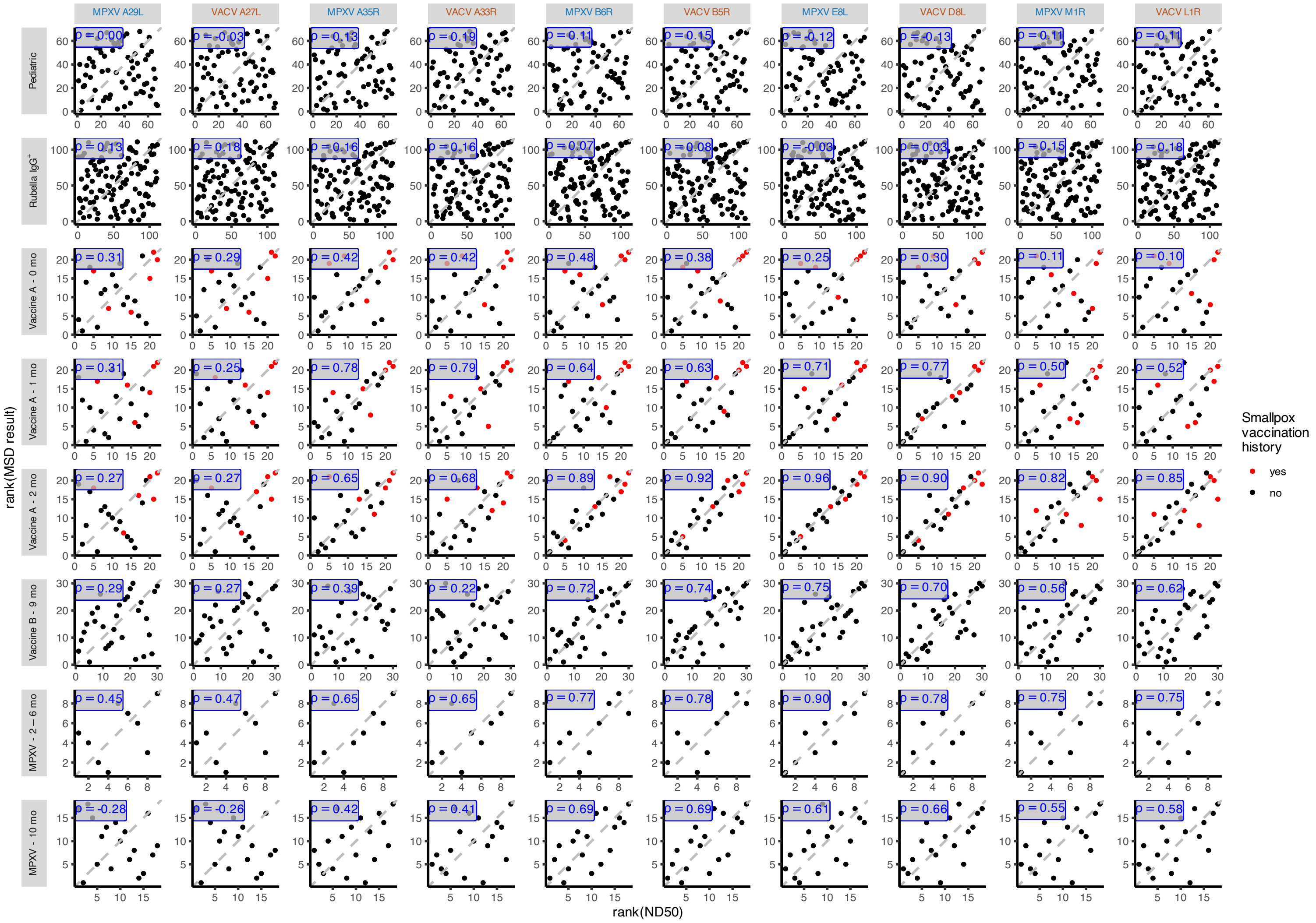

Figure S11: The anti-orthopoxvirus antibody titer results and MVA neutralization results are generally correlated in sera from individuals know to be MPXV infected or vaccinated. Shown are pairwise plots of the rank ordered MSD AU/mL results (MSD Response) verses rank ordered MVA neutralization assay ND50 results. Inset value in blue is the Spearman's correlation coefficient, also shown in Figure 4B. Results are filled with red if specimen is from an individual with known smallpox vaccination history.

medRxiv preprint doi: https://doi.org/10.1101/2025.10.24.25338410; this version posted October 27, 2025. The copyright holder for this preprint (which was not certified by peer review) is the author/funder, who has granted medRxiv a license to display the preprint in perpetuity.
It is made available under a CC-BY 4.0 International license .

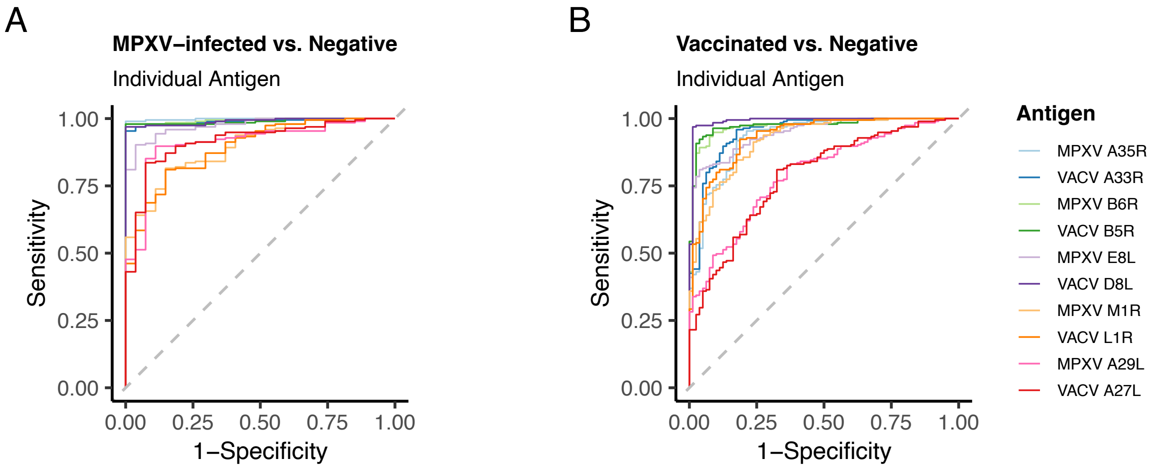

Figure S12: Receiver operator characteristic (ROC) curves separately comparing each exposed group (MPXV-infected or vaccinated) to negative controls. MPXV-infected refer to all sera from known MPXV infected individuals, Vaccinated refers to all sera from known vaccinated individuals, and Negative refer to all sera from the rubella and pediatric negative cohorts combined. Baseline sera collected prior to vaccination (Vaccine A – 0 month) were included in the Vaccinated group if they had a history of smallpox vaccination, otherwise they were categorized as negative (6 out of 22 individuals had previous history of vaccination). (A) ROC curves for all individual antigens generated by comparing all known MPXV-infected (n = 27) to all negatives (n = 195). (B) ROC curves for all individual antigens generated by comparing all known Vaccinated (n = 80) to all to all negatives (n = 195). Summary statistics from this analysis can be found Tables S5-S6.

medRxiv preprint doi: https://doi.org/10.1101/2025.10.24.25338410; this version posted October 27, 2025. The copyright holder for this preprint (which was not certified by peer review) is the author/funder, who has granted medRxiv a license to display the preprint in perpetuity.
It is made available under a CC-BY 4.0 International license .

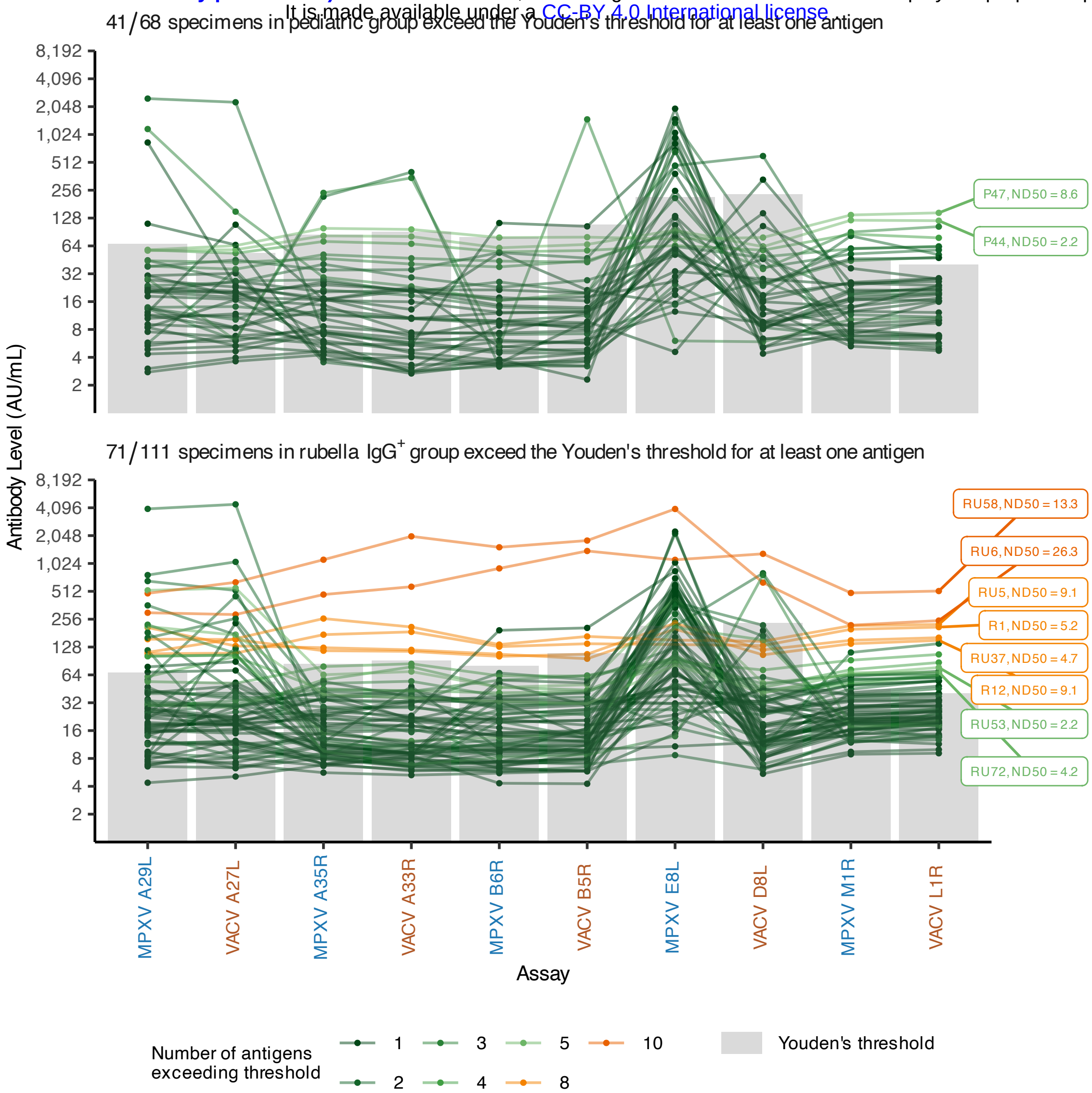

Figure S13: Some specimens in the negative cohorts (pediatric and rubella IgG+) exceed Youden's threshold (based on distinguishing exposed and unexposed groups) for one or more antigens. For each negative cohort (upper, pediatric; lower, rubella IgG+) the antibody level (AU/mL) is plotted for specimens that exceed Youden's threshold based on distinguish exposed vs. unexposed groups for at least one antigen (see Table S5). Specimens that exceeded the threshold for five or more antigens are labeled with the specimen identifier and MVA-neutralization ND50.
